# 11-[3-(Dimethyl­amino)prop­yl]-6,11-dihydro­dibenzo[*b*,*e*]thiepin-11-ol

**DOI:** 10.1107/S1600536809053434

**Published:** 2009-12-16

**Authors:** Jerry P. Jasinski, Ray J. Butcher, Q. N. M. Hakim Al-arique, H. S. Yathirajan, A.R. Ramesha

**Affiliations:** a Department of Chemistry, Keene State College, 229 Main Street, Keene, NH 03435-2001, USA; b Department of Chemistry, Howard University, 525 College Street NW, Washington, DC 20059, USA; c Department of Studies in Chemistry, University of Mysore, Manasagangotri, 570 006, India; d RL Fine Chem, Bangalore, 560 064, India

## Abstract

There are two independent mol­ecules (*A* and *B*) in the asymmetric unit of the title compound, C_19_H_23_NOS. In each mol­ecule, the seven-membered thiepine ring is bent into a slightly twisted V-shape. The dihedral angles between the mean planes of the two benzene rings fused to the thiepine ring are 75.7 (5) in mol­ecule *A* and 73.8 (4)° in mol­ecule *B*. In both mol­ecules, an intra­molecular O—H⋯N hydrogen bond occurs. In the crystal, weak inter­molecular C—H⋯O and C—H⋯π-ring inter­actions are observed.

## Related literature

For related structures, see: Bandoli & Nicolini, (1982 ▶); Blaton *et al.* (1995 ▶); Ieawsuwan *et al.* (2006 ▶); Linden *et al.* (2004 ▶); Portalone *et al.* (2007 ▶); Roszak *et al.* (1996 ▶); Rudorf *et al.* (1999 ▶); Yoshinari & Konno, (2009 ▶); Zhang *et al.* (2008 ▶,2008*a* ▶). For related background, see: Rudorf *et al.* (1999 ▶). For antidepressant and anti-inflammatory properties, see: Rajsner *et al.* (1969 ▶, 1971 ▶); Rooks *et al.* (1980 ▶); Tomascovic *et al.* (2000 ▶); Truce *et al.* (1956 ▶). For pharmacological synthesis and studies, see: Ikuo *et al.* (1978 ▶); Uchida *et al.* (1979 ▶); Wyatt *et al.* (2006 ▶). For NMR, Ir and X-ray studies, see: Kolehmainen *et al.* (2007 ▶). For density functional theory (DFT), see: Becke (1988 ▶, 1993 ▶); Frisch *et al.* (2004 ▶); Hehre *et al.* (1986 ▶); Lee *et al.* (1988 ▶); Schmidt & Polik (2007 ▶).
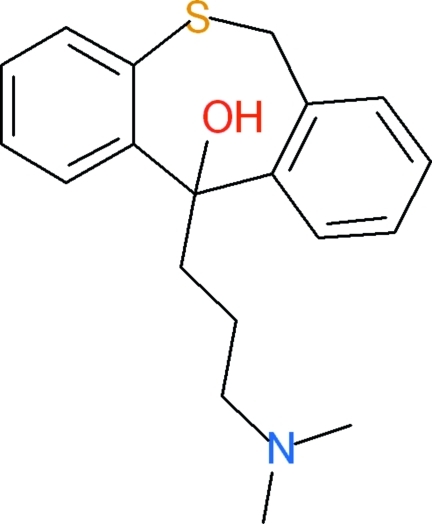


## Experimental

### 

#### Crystal data

C_19_H_23_NOS
*M* *_r_* = 313.44
Monoclinic, 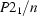

*a* = 7.7215 (4) Å
*b* = 15.3729 (10) Å
*c* = 27.9274 (16) Å
β = 95.401 (6)°
*V* = 3300.3 (3) Å^3^
*Z* = 8
Cu *K*α radiation
μ = 1.74 mm^−1^
*T* = 110 K
0.51 × 0.42 × 0.14 mm

#### Data collection

Oxford Diffraction Xcalibur diffractometer with a Ruby (Gemini Cu) detector
Absorption correction: multi-scan (*CrysAlis RED*; Oxford Diffraction, 2007 ▶) *T*_min_ = 0.432, *T*_max_ = 1.000
14666 measured reflections
6565 independent reflections
5490 reflections with *I* > 2σ(*I*)
*R*_int_ = 0.029

#### Refinement

*R*[*F* ^2^ > 2σ(*F* ^2^)] = 0.054
*wR*(*F* ^2^) = 0.153
*S* = 1.05
6565 reflections
403 parameters
H-atom parameters constrained
Δρ_max_ = 0.58 e Å^−3^
Δρ_min_ = −0.56 e Å^−3^

### 

Data collection: *CrysAlis PRO* (Oxford Diffraction, 2007 ▶); cell refinement: *CrysAlis RED* (Oxford Diffraction, 2007 ▶); data reduction: *CrysAlis RED*; program(s) used to solve structure: *SHELXS97* (Sheldrick, 2008 ▶); program(s) used to refine structure: *SHELXL97* (Sheldrick, 2008 ▶); molecular graphics: *SHELXTL* (Sheldrick, 2008 ▶); software used to prepare material for publication: *SHELXTL* (Sheldrick, 2008 ▶).

## Supplementary Material

Crystal structure: contains datablocks global, I. DOI: 10.1107/S1600536809053434/lh2968sup1.cif

Structure factors: contains datablocks I. DOI: 10.1107/S1600536809053434/lh2968Isup2.hkl

Additional supplementary materials:  crystallographic information; 3D view; checkCIF report

## Figures and Tables

**Table 1:** Hydrogen-bond geometry (Å, °)

*D*—H⋯*A*	*D*—H	H⋯*A*	*D*⋯*A*	*D*—H⋯*A*
O1*A*—H1*A*⋯N1*A*	0.84	1.86	2.693 (2)	170
O1*B*—H1*B*⋯N1*B*	0.84	1.84	2.679 (2)	174
C4*A*—H4*AA*⋯O1*B*	0.95	2.51	3.253 (2)	135
C3*A*—H3*AA*⋯*Cg*7^i^	0.95	2.74	3.526 (6)	140
C17*A*—H17*A*⋯*Cg*1^ii^	0.99	2.67	3.537 (7)	147
C17*A*—H17*B*⋯*Cg*2^ii^	0.99	2.75	3.720 (3)	167
C17*B*—H17*C*⋯*Cg*8^iii^	0.99	2.68	3.663 (6)	170
C17*B*—17D⋯*Cg*7^iii^	0.99	2.64	3.538 (1)	149

Symmetry codes: (i) 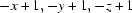; (ii) 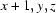; (iii) 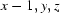. *Cg*1, *Cg*2, *Cg*7 and *Cg*8 are the centroids of the C1*A*—C6*A*, C8*A*—C13*A*, C1*B*–C6*B* and C8*B*—C13*B* rings, respectively.

**Fig. 1.**
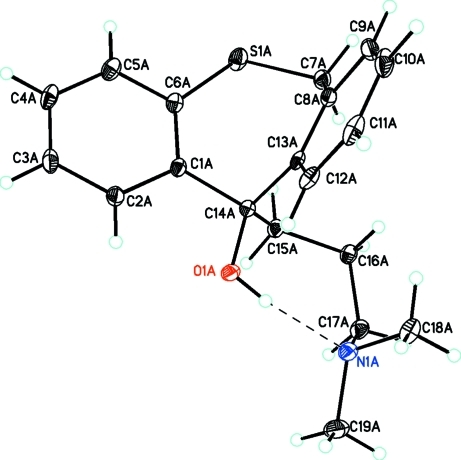
Molecular structure of molecule A in (I) showing the atom labeling scheme and 50% probability displacement ellipsoids.

**Fig. 2.**
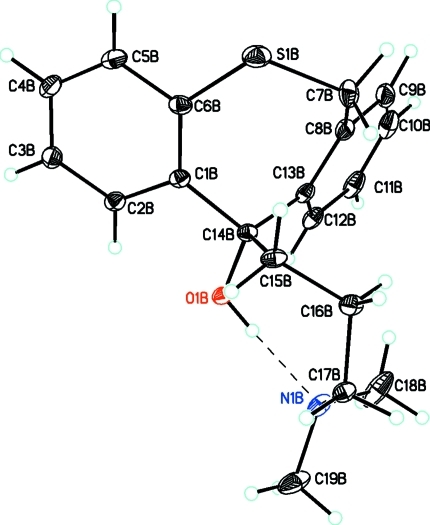
Molecular structure of molecule B in (I) showing the atom labeling scheme and 50% probability displacement ellipsoids.

**Fig. 3.**
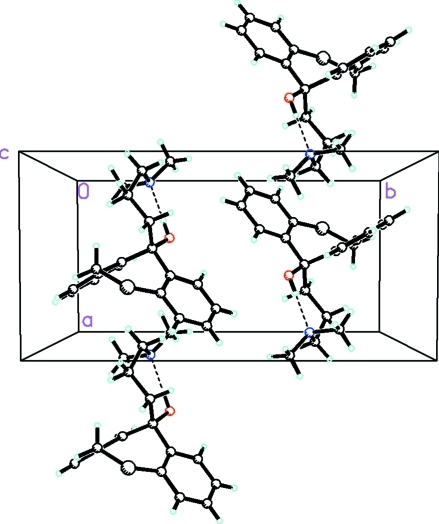
Packing diagram of (I), viewed along the c axis. Dashed lines indicate O—H···N intramolecular interactions in molecules A & B.

## supplementary crystallographic information

### Comment

The title compound, (I), C_19_H_23_NOS, is a derivative of 6,11-dihydrodibenzo[b,e]thiepin-11-one, which is used as an intermediate for the synthesis of dosulepin, an antidepressant of the tricyclic family. The dibenzo[c,e]thiepine derivatives (Truce *et al.*, 1956) exhibit remarkable chiroptical properties (Tomascovic *et al.*, 2000). The anti-inflammatory and analgesic profile of 6,11-dihydrodibenzo[b,e]thiepin-11-one-3-acetic acid (Tiopinac) is reported (Rooks II *et al.*, 1980). Dibenzo[b,e]thiepin-5,5-dioxide derivatives are known to possess antihistaminic and antiallergenic activities (Rajsner *et al.*, 1971). In addition, by aminoalkylation of 6,11-dihydrodibenzo[b,e]thiepin-5,5-dioxide and the corresponding 11-ketone, compounds with neurotropic and psychotropic activities have been reported (Rajsner *et al.*, 1969). Also, the comparative NMR and IR spectral, X-ray structural and theoretical studies of eight 6-arylidenedibenzo[b,e]thiepin-11-one-5,5-dioxides have been reported (Kolehmainen *et al.*, 2007). A pharmacological study of [2-chloro-11-(2-dimethylaminoethoxy)dibenzo(b,f)thiepine] (zotepine), and a new neuroleptic drug are also reported (Uchida *et al.*, 1979). In addition, the synthesis and chemistry of enantiomerically pure 10,11-dihydrobenzo[b,f]thiepines (Wyatt *et al.*, 2006) and the synthesis and pharmacological properties of 8-chloro-10-(2-dimethylaminoethoxy) dibenzo[b,f]thiepine and related compounds have been reported (Ikuo *et al.*, 1978). In view of the importance of thiepines, this paper reports the crystal structure of the title compound, C_19_H_23_NOS, (I).

The title compound, C_19_H_23_NOS, (I), crystallizes with two independent molecules (A, Fig. 1 & B, Fig. 2) in the asymmetric unit. The seven-membered thiepine ring is bent into a slightly twisted V-shaped arrangement with sp3 hybridized atoms at C7(A & B), C14(A & B)and S1(A & B). The dihedral angles between the mean planes of the two benzene rings fused to the thiepine ring are 75.7 (5)° (A) and 73.8 (4)° (B), respectively. An intramolecular O—H···N hydrogen bond exists between the hydroxy group and the N atom from the (dimethylamino)propyl group both bonded to the C14 atom of the thiepine ring (O1A—H1A···N1A & O1B—H1B···N1; Table 1). While no classical intermolecular hydrogen bonds are present, weak C–H···O and C–H···π-ring intermolecular interactions are observed which contribute to the stability of crystal packing (Fig.3, Table 1,2).

Following a geometry optimization density functional theory calculation (Schmidt & Polik 2007) at the B3LYP 6–31-G(*d*) level (Becke, 1988, 1993; Lee *et al.* 1988; Hehre *et al.* 1986) with the Gaussian03 program package (Frisch *at al.* 2004) the angle between the mean planes of the two benzene rings changes to 73.4 (4)°, a difference of -2.32° (A) and + 0.40° (B), respectively. These results support the collective effects of the intra and intermolecular hydrogen bonding described above slightly influencing crystal packing.

### Experimental

The title compound was obtained as a gift sample from *R. L*. Fine Chem, Bangalore, India. The compound was used without further purification. X-ray quality crystals (m.p. 433–435 K) of the title compound, (I), were obtained by slow evaporation from acetone solution.

### Refinement

The hydroxy H atoms, H1A and H1B, were found in a difference map and refined freely. All of the C-bonded H atoms were placed in their calculated positions and then refined using the riding model with C—H = 0.95 to 0.99 Å, and with *U*_iso_(H) = 1.18–1.50 *U*_eq_(C). Methyl groups were allowed to rotate about their N—C bonds.

### Crystal data

**Table:**

C_19_H_23_NOS	*F*(000) = 1344
*M**_r_* = 313.44	*D*_x_ = 1.262 Mg m^−^^3^
Monoclinic, *P*2_1_/*n*	Cu *K*α radiation, λ = 1.54184 Å
Hall symbol: -P 2yn	Cell parameters from 6805 reflections
*a* = 7.7215 (4) Å	θ = 4.3–74.0°
*b* = 15.3729 (10) Å	µ = 1.74 mm^−^^1^
*c* = 27.9274 (16) Å	*T* = 110 K
β = 95.401 (6)°	Plate, colorless
*V* = 3300.3 (3) Å^3^	0.51 × 0.42 × 0.14 mm
*Z* = 8	

### Data collection

**Table:**

Oxford Diffraction Xcalibur diffractometer with a Ruby (Gemini Cu) detector	6565 independent reflections
Radiation source: Enhance (Cu) X-ray Source	5490 reflections with *I* > 2σ(*I*)
graphite	*R*_int_ = 0.029
Detector resolution: 10.5081 pixels mm^-1^	θ_max_ = 74.2°, θ_min_ = 4.3°
ω scans	*h* = −9→9
Absorption correction: multi-scan (*CrysAlis RED*; Oxford Diffraction, 2007)	*k* = −13→19
*T*_min_ = 0.432, *T*_max_ = 1.000	*l* = −17→34
14666 measured reflections	

### Refinement

**Table:**

Refinement on *F*^2^	Primary atom site location: structure-invariant direct methods
Least-squares matrix: full	Secondary atom site location: difference Fourier map
*R*[*F*^2^ > 2σ(*F*^2^)] = 0.054	Hydrogen site location: inferred from neighbouring sites
*wR*(*F*^2^) = 0.153	H-atom parameters constrained
*S* = 1.05	*w* = 1/[σ^2^(*F*_o_^2^) + (0.1079*P*)^2^ + 0.6589*P*] where *P* = (*F*_o_^2^ + 2*F*_c_^2^)/3
6565 reflections	(Δ/σ)_max_ = 0.001
403 parameters	Δρ_max_ = 0.57 e Å^−^^3^
0 restraints	Δρ_min_ = −0.56 e Å^−^^3^

### Special details

**Table:**

Geometry. All e.s.d.'s (except the e.s.d. in the dihedral angle between two l.s. planes) are estimated using the full covariance matrix. The cell e.s.d.'s are taken into account individually in the estimation of e.s.d.'s in distances, angles and torsion angles; correlations between e.s.d.'s in cell parameters are only used when they are defined by crystal symmetry. An approximate (isotropic) treatment of cell e.s.d.'s is used for estimating e.s.d.'s involving l.s. planes.
Refinement. Refinement of *F*^2^ against ALL reflections. The weighted *R*-factor *wR* and goodness of fit *S* are based on *F*^2^, conventional *R*-factors *R* are based on *F*, with *F* set to zero for negative *F*^2^. The threshold expression of *F*^2^ > σ(*F*^2^) is used only for calculating *R*-factors(gt) *etc*. and is not relevant to the choice of reflections for refinement. *R*-factors based on *F*^2^ are statistically about twice as large as those based on *F*, and *R*- factors based on ALL data will be even larger.

### Fractional atomic coordinates and isotropic or equivalent isotropic displacement parameters (Å^2^)

**Table:**

	*x*	*y*	*z*	*U*_iso_*/*U*_eq_	
S1A	0.69626 (7)	0.25999 (3)	0.288172 (19)	0.02978 (15)	
O1A	0.91923 (17)	0.54941 (9)	0.28401 (5)	0.0240 (3)	
H1A	1.0075	0.5603	0.2696	0.029*	
N1A	1.2061 (2)	0.56334 (11)	0.23664 (6)	0.0242 (4)	
C1A	0.7385 (2)	0.44069 (13)	0.31337 (6)	0.0189 (4)	
C2A	0.6889 (2)	0.50798 (13)	0.34289 (6)	0.0230 (4)	
H2AA	0.7418	0.5635	0.3408	0.028*	
C3A	0.5643 (3)	0.49613 (15)	0.37524 (7)	0.0276 (4)	
H3AA	0.5350	0.5429	0.3952	0.033*	
C4A	0.4833 (3)	0.41658 (15)	0.37828 (7)	0.0284 (4)	
H4AA	0.3984	0.4079	0.4003	0.034*	
C5A	0.5275 (3)	0.34982 (14)	0.34876 (7)	0.0264 (4)	
H5AA	0.4697	0.2955	0.3502	0.032*	
C6A	0.6554 (2)	0.35985 (13)	0.31669 (6)	0.0202 (4)	
C7A	0.7862 (3)	0.28132 (13)	0.23129 (7)	0.0238 (4)	
H7AA	0.9147	0.2814	0.2370	0.029*	
H7AB	0.7527	0.2330	0.2089	0.029*	
C8A	0.7295 (2)	0.36527 (13)	0.20746 (6)	0.0202 (4)	
C9A	0.6275 (3)	0.36079 (15)	0.16343 (7)	0.0280 (4)	
H9AA	0.5927	0.3055	0.1508	0.034*	
C10A	0.5764 (3)	0.43455 (17)	0.13802 (7)	0.0331 (5)	
H10A	0.5075	0.4301	0.1081	0.040*	
C11A	0.6263 (3)	0.51514 (16)	0.15646 (7)	0.0306 (5)	
H11A	0.5926	0.5665	0.1391	0.037*	
C12A	0.7258 (2)	0.52091 (13)	0.20050 (7)	0.0232 (4)	
H12A	0.7588	0.5766	0.2129	0.028*	
C13A	0.7782 (2)	0.44720 (12)	0.22683 (6)	0.0170 (4)	
C14A	0.8737 (2)	0.46053 (12)	0.27754 (6)	0.0181 (4)	
C15A	1.0403 (2)	0.40533 (13)	0.28923 (6)	0.0207 (4)	
H15A	1.1021	0.4278	0.3194	0.025*	
H15B	1.0046	0.3448	0.2955	0.025*	
C16A	1.1698 (2)	0.40312 (13)	0.25052 (7)	0.0218 (4)	
H16A	1.2402	0.3494	0.2550	0.026*	
H16B	1.1027	0.3994	0.2186	0.026*	
C17A	1.2933 (2)	0.48049 (13)	0.25020 (7)	0.0241 (4)	
H17A	1.3558	0.4868	0.2826	0.029*	
H17B	1.3810	0.4683	0.2274	0.029*	
C18A	1.1570 (3)	0.56816 (15)	0.18463 (7)	0.0296 (5)	
H18A	1.0974	0.6234	0.1769	0.044*	
H18B	1.0791	0.5197	0.1748	0.044*	
H18C	1.2618	0.5646	0.1675	0.044*	
C19A	1.3169 (3)	0.63736 (16)	0.25201 (10)	0.0396 (6)	
H19A	1.2540	0.6917	0.2442	0.059*	
H19B	1.4230	0.6358	0.2353	0.059*	
H19C	1.3477	0.6342	0.2868	0.059*	
S1B	0.66729 (7)	0.22246 (4)	0.627252 (18)	0.03356 (16)	
O1B	0.39362 (17)	0.32974 (9)	0.47928 (5)	0.0224 (3)	
H1B	0.2941	0.3119	0.4691	0.027*	
N1B	0.0713 (2)	0.27183 (13)	0.45327 (6)	0.0272 (4)	
C1B	0.6076 (2)	0.33566 (12)	0.54588 (6)	0.0194 (4)	
C2B	0.6575 (2)	0.41167 (13)	0.52343 (7)	0.0225 (4)	
H2BA	0.5966	0.4284	0.4937	0.027*	
C3B	0.7936 (3)	0.46361 (14)	0.54324 (8)	0.0269 (4)	
H3BA	0.8237	0.5154	0.5274	0.032*	
C4B	0.8849 (3)	0.43913 (14)	0.58631 (8)	0.0296 (5)	
H4BA	0.9773	0.4743	0.6003	0.036*	
C5B	0.8406 (3)	0.36365 (14)	0.60857 (7)	0.0279 (4)	
H5BA	0.9048	0.3467	0.6378	0.033*	
C6B	0.7032 (2)	0.31114 (13)	0.58920 (7)	0.0227 (4)	
C7B	0.5684 (3)	0.13299 (14)	0.59244 (7)	0.0292 (4)	
H7BA	0.6145	0.0780	0.6070	0.035*	
H7BB	0.4418	0.1340	0.5955	0.035*	
C8B	0.5951 (2)	0.13126 (13)	0.53919 (7)	0.0249 (4)	
C9B	0.6839 (3)	0.05921 (14)	0.52276 (9)	0.0340 (5)	
H9BA	0.7316	0.0173	0.5453	0.041*	
C10B	0.7035 (3)	0.04778 (15)	0.47439 (10)	0.0382 (6)	
H10B	0.7637	−0.0015	0.4638	0.046*	
C11B	0.6349 (3)	0.10843 (16)	0.44183 (8)	0.0341 (5)	
H11B	0.6440	0.1002	0.4084	0.041*	
C12B	0.5524 (2)	0.18169 (14)	0.45762 (7)	0.0257 (4)	
H12B	0.5087	0.2240	0.4348	0.031*	
C13B	0.5316 (2)	0.19501 (13)	0.50640 (7)	0.0199 (4)	
C14B	0.4538 (2)	0.28306 (12)	0.52111 (6)	0.0187 (4)	
C15B	0.3036 (2)	0.27781 (14)	0.55385 (7)	0.0231 (4)	
H15C	0.3525	0.2592	0.5863	0.028*	
H15D	0.2554	0.3370	0.5570	0.028*	
C16B	0.1534 (2)	0.21639 (14)	0.53708 (7)	0.0258 (4)	
H16C	0.2030	0.1642	0.5227	0.031*	
H16D	0.0976	0.1970	0.5657	0.031*	
C17B	0.0125 (2)	0.25346 (13)	0.50080 (7)	0.0220 (4)	
H17C	−0.0853	0.2116	0.4968	0.026*	
H17D	−0.0321	0.3080	0.5140	0.026*	
C18B	0.0944 (3)	0.1914 (2)	0.42672 (9)	0.0515 (8)	
H18D	0.1890	0.1573	0.4433	0.077*	
H18E	0.1232	0.2054	0.3942	0.077*	
H18F	−0.0136	0.1575	0.4248	0.077*	
C19B	−0.0530 (3)	0.3298 (2)	0.42629 (9)	0.0479 (7)	
H19D	−0.0118	0.3430	0.3950	0.072*	
H19E	−0.0635	0.3839	0.4444	0.072*	
H19F	−0.1668	0.3012	0.4215	0.072*	

### Atomic displacement parameters (Å^2^)

**Table:**

	*U*^11^	*U*^22^	*U*^33^	*U*^12^	*U*^13^	*U*^23^
S1A	0.0386 (3)	0.0189 (2)	0.0344 (3)	−0.0007 (2)	0.0165 (2)	0.00348 (19)
O1A	0.0191 (7)	0.0210 (7)	0.0333 (7)	−0.0042 (5)	0.0094 (5)	−0.0068 (6)
N1A	0.0208 (8)	0.0233 (8)	0.0293 (8)	−0.0028 (6)	0.0070 (6)	−0.0039 (7)
C1A	0.0150 (8)	0.0269 (9)	0.0148 (8)	0.0016 (7)	0.0015 (6)	0.0003 (7)
C2A	0.0194 (9)	0.0291 (10)	0.0208 (8)	0.0002 (7)	0.0025 (7)	−0.0039 (8)
C3A	0.0247 (10)	0.0398 (12)	0.0188 (8)	0.0059 (9)	0.0047 (7)	−0.0055 (8)
C4A	0.0240 (10)	0.0434 (12)	0.0189 (8)	0.0055 (9)	0.0078 (7)	0.0078 (8)
C5A	0.0249 (10)	0.0315 (11)	0.0234 (9)	0.0026 (8)	0.0059 (7)	0.0092 (8)
C6A	0.0209 (9)	0.0222 (9)	0.0177 (8)	0.0033 (7)	0.0022 (7)	0.0030 (7)
C7A	0.0269 (10)	0.0198 (9)	0.0258 (9)	0.0003 (7)	0.0088 (7)	−0.0037 (7)
C8A	0.0184 (9)	0.0251 (10)	0.0177 (8)	−0.0006 (7)	0.0051 (7)	−0.0007 (7)
C9A	0.0237 (10)	0.0395 (12)	0.0210 (9)	−0.0004 (9)	0.0031 (7)	−0.0090 (8)
C10A	0.0236 (10)	0.0594 (15)	0.0164 (8)	0.0080 (10)	0.0018 (7)	0.0004 (9)
C11A	0.0210 (10)	0.0468 (13)	0.0254 (9)	0.0101 (9)	0.0091 (7)	0.0159 (9)
C12A	0.0166 (8)	0.0265 (10)	0.0277 (9)	0.0028 (7)	0.0089 (7)	0.0068 (8)
C13A	0.0121 (8)	0.0230 (9)	0.0169 (8)	0.0016 (7)	0.0065 (6)	0.0015 (7)
C14A	0.0166 (8)	0.0192 (9)	0.0190 (8)	−0.0008 (7)	0.0041 (6)	−0.0021 (7)
C15A	0.0168 (8)	0.0257 (10)	0.0198 (8)	0.0013 (7)	0.0026 (6)	−0.0003 (7)
C16A	0.0171 (9)	0.0246 (9)	0.0242 (9)	0.0036 (7)	0.0049 (7)	0.0001 (7)
C17A	0.0160 (8)	0.0306 (10)	0.0260 (9)	0.0008 (8)	0.0033 (7)	−0.0002 (8)
C18A	0.0264 (10)	0.0345 (11)	0.0293 (10)	0.0043 (8)	0.0103 (8)	0.0074 (9)
C19A	0.0314 (12)	0.0322 (12)	0.0572 (14)	−0.0121 (10)	0.0155 (11)	−0.0117 (11)
S1B	0.0336 (3)	0.0426 (3)	0.0233 (3)	0.0001 (2)	−0.00317 (19)	0.0080 (2)
O1B	0.0184 (6)	0.0281 (7)	0.0199 (6)	−0.0033 (5)	−0.0022 (5)	0.0059 (5)
N1B	0.0221 (8)	0.0414 (10)	0.0180 (7)	−0.0099 (7)	0.0010 (6)	0.0005 (7)
C1B	0.0158 (8)	0.0226 (9)	0.0200 (8)	0.0041 (7)	0.0026 (7)	−0.0039 (7)
C2B	0.0179 (9)	0.0246 (9)	0.0248 (9)	0.0030 (7)	0.0018 (7)	−0.0044 (8)
C3B	0.0213 (9)	0.0237 (9)	0.0357 (10)	0.0016 (8)	0.0033 (8)	−0.0048 (8)
C4B	0.0206 (9)	0.0308 (11)	0.0362 (11)	0.0004 (8)	−0.0034 (8)	−0.0133 (9)
C5B	0.0222 (10)	0.0354 (11)	0.0250 (9)	0.0063 (8)	−0.0038 (7)	−0.0070 (8)
C6B	0.0213 (9)	0.0254 (9)	0.0212 (8)	0.0062 (7)	0.0014 (7)	−0.0019 (8)
C7B	0.0353 (11)	0.0253 (10)	0.0283 (10)	−0.0100 (9)	0.0094 (8)	0.0047 (8)
C8B	0.0171 (9)	0.0241 (10)	0.0336 (10)	−0.0039 (7)	0.0027 (7)	0.0009 (8)
C9B	0.0218 (10)	0.0223 (10)	0.0581 (14)	−0.0020 (8)	0.0040 (9)	0.0019 (10)
C10B	0.0218 (10)	0.0288 (11)	0.0657 (16)	−0.0039 (9)	0.0135 (10)	−0.0190 (11)
C11B	0.0229 (10)	0.0411 (12)	0.0403 (11)	−0.0105 (9)	0.0127 (9)	−0.0188 (10)
C12B	0.0175 (9)	0.0341 (11)	0.0264 (9)	−0.0067 (8)	0.0061 (7)	−0.0057 (8)
C13B	0.0117 (8)	0.0243 (9)	0.0241 (9)	−0.0032 (7)	0.0045 (6)	−0.0036 (7)
C14B	0.0169 (9)	0.0228 (9)	0.0163 (8)	0.0010 (7)	0.0014 (6)	0.0015 (7)
C15B	0.0168 (9)	0.0341 (10)	0.0184 (8)	0.0037 (8)	0.0022 (7)	0.0015 (8)
C16B	0.0178 (9)	0.0327 (11)	0.0274 (9)	−0.0008 (8)	0.0048 (7)	0.0102 (8)
C17B	0.0169 (9)	0.0289 (10)	0.0206 (8)	−0.0005 (7)	0.0038 (7)	−0.0008 (7)
C18B	0.0343 (13)	0.079 (2)	0.0434 (13)	−0.0256 (13)	0.0172 (11)	−0.0361 (14)
C19B	0.0280 (12)	0.0747 (19)	0.0382 (12)	−0.0149 (12)	−0.0115 (9)	0.0277 (13)

### Geometric parameters (Å, °)

**Table:**

S1A—C6A	1.7713 (19)	S1B—C6B	1.766 (2)
S1A—C7A	1.822 (2)	S1B—C7B	1.811 (2)
O1A—C14A	1.418 (2)	O1B—C14B	1.412 (2)
O1A—H1A	0.8400	O1B—H1B	0.8400
N1A—C19A	1.463 (3)	N1B—C18B	1.461 (3)
N1A—C18A	1.468 (3)	N1B—C19B	1.465 (3)
N1A—C17A	1.473 (3)	N1B—C17B	1.470 (2)
C1A—C2A	1.398 (3)	C1B—C2B	1.397 (3)
C1A—C6A	1.406 (3)	C1B—C6B	1.408 (2)
C1A—C14A	1.543 (2)	C1B—C14B	1.545 (2)
C2A—C3A	1.393 (3)	C2B—C3B	1.393 (3)
C2A—H2AA	0.9500	C2B—H2BA	0.9500
C3A—C4A	1.380 (3)	C3B—C4B	1.388 (3)
C3A—H3AA	0.9500	C3B—H3BA	0.9500
C4A—C5A	1.380 (3)	C4B—C5B	1.375 (3)
C4A—H4AA	0.9500	C4B—H4BA	0.9500
C5A—C6A	1.402 (3)	C5B—C6B	1.401 (3)
C5A—H5AA	0.9500	C5B—H5BA	0.9500
C7A—C8A	1.498 (3)	C7B—C8B	1.521 (3)
C7A—H7AA	0.9900	C7B—H7BA	0.9900
C7A—H7AB	0.9900	C7B—H7BB	0.9900
C8A—C9A	1.398 (3)	C8B—C13B	1.398 (3)
C8A—C13A	1.408 (3)	C8B—C9B	1.402 (3)
C9A—C10A	1.376 (3)	C9B—C10B	1.384 (4)
C9A—H9AA	0.9500	C9B—H9BA	0.9500
C10A—C11A	1.382 (3)	C10B—C11B	1.373 (4)
C10A—H10A	0.9500	C10B—H10B	0.9500
C11A—C12A	1.390 (3)	C11B—C12B	1.386 (3)
C11A—H11A	0.9500	C11B—H11B	0.9500
C12A—C13A	1.390 (3)	C12B—C13B	1.402 (3)
C12A—H12A	0.9500	C12B—H12B	0.9500
C13A—C14A	1.548 (2)	C13B—C14B	1.552 (3)
C14A—C15A	1.550 (2)	C14B—C15B	1.545 (2)
C15A—C16A	1.540 (2)	C15B—C16B	1.534 (3)
C15A—H15A	0.9900	C15B—H15C	0.9900
C15A—H15B	0.9900	C15B—H15D	0.9900
C16A—C17A	1.525 (3)	C16B—C17B	1.525 (3)
C16A—H16A	0.9900	C16B—H16C	0.9900
C16A—H16B	0.9900	C16B—H16D	0.9900
C17A—H17A	0.9900	C17B—H17C	0.9900
C17A—H17B	0.9900	C17B—H17D	0.9900
C18A—H18A	0.9800	C18B—H18D	0.9800
C18A—H18B	0.9800	C18B—H18E	0.9800
C18A—H18C	0.9800	C18B—H18F	0.9800
C19A—H19A	0.9800	C19B—H19D	0.9800
C19A—H19B	0.9800	C19B—H19E	0.9800
C19A—H19C	0.9800	C19B—H19F	0.9800
			
C6A—S1A—C7A	109.55 (9)	C6B—S1B—C7B	110.20 (9)
C14A—O1A—H1A	109.5	C14B—O1B—H1B	109.5
C19A—N1A—C18A	109.89 (18)	C18B—N1B—C19B	111.0 (2)
C19A—N1A—C17A	110.88 (17)	C18B—N1B—C17B	111.00 (19)
C18A—N1A—C17A	111.53 (16)	C19B—N1B—C17B	109.77 (18)
C2A—C1A—C6A	117.58 (17)	C2B—C1B—C6B	117.73 (17)
C2A—C1A—C14A	118.41 (17)	C2B—C1B—C14B	118.00 (15)
C6A—C1A—C14A	123.95 (16)	C6B—C1B—C14B	124.27 (17)
C3A—C2A—C1A	122.01 (19)	C3B—C2B—C1B	122.07 (18)
C3A—C2A—H2AA	119.0	C3B—C2B—H2BA	119.0
C1A—C2A—H2AA	119.0	C1B—C2B—H2BA	119.0
C4A—C3A—C2A	120.05 (19)	C4B—C3B—C2B	119.4 (2)
C4A—C3A—H3AA	120.0	C4B—C3B—H3BA	120.3
C2A—C3A—H3AA	120.0	C2B—C3B—H3BA	120.3
C5A—C4A—C3A	118.91 (18)	C5B—C4B—C3B	119.56 (19)
C5A—C4A—H4AA	120.5	C5B—C4B—H4BA	120.2
C3A—C4A—H4AA	120.5	C3B—C4B—H4BA	120.2
C4A—C5A—C6A	121.9 (2)	C4B—C5B—C6B	121.55 (18)
C4A—C5A—H5AA	119.0	C4B—C5B—H5BA	119.2
C6A—C5A—H5AA	119.0	C6B—C5B—H5BA	119.2
C5A—C6A—C1A	119.50 (18)	C5B—C6B—C1B	119.62 (19)
C5A—C6A—S1A	110.96 (15)	C5B—C6B—S1B	111.62 (14)
C1A—C6A—S1A	129.40 (14)	C1B—C6B—S1B	128.64 (16)
C8A—C7A—S1A	115.01 (13)	C8B—C7B—S1B	116.69 (14)
C8A—C7A—H7AA	108.5	C8B—C7B—H7BA	108.1
S1A—C7A—H7AA	108.5	S1B—C7B—H7BA	108.1
C8A—C7A—H7AB	108.5	C8B—C7B—H7BB	108.1
S1A—C7A—H7AB	108.5	S1B—C7B—H7BB	108.1
H7AA—C7A—H7AB	107.5	H7BA—C7B—H7BB	107.3
C9A—C8A—C13A	119.34 (18)	C13B—C8B—C9B	119.4 (2)
C9A—C8A—C7A	117.71 (18)	C13B—C8B—C7B	123.82 (18)
C13A—C8A—C7A	122.93 (16)	C9B—C8B—C7B	116.7 (2)
C10A—C9A—C8A	121.6 (2)	C10B—C9B—C8B	121.4 (2)
C10A—C9A—H9AA	119.2	C10B—C9B—H9BA	119.3
C8A—C9A—H9AA	119.2	C8B—C9B—H9BA	119.3
C9A—C10A—C11A	119.35 (18)	C11B—C10B—C9B	119.3 (2)
C9A—C10A—H10A	120.3	C11B—C10B—H10B	120.3
C11A—C10A—H10A	120.3	C9B—C10B—H10B	120.3
C10A—C11A—C12A	119.9 (2)	C10B—C11B—C12B	120.1 (2)
C10A—C11A—H11A	120.1	C10B—C11B—H11B	120.0
C12A—C11A—H11A	120.1	C12B—C11B—H11B	120.0
C13A—C12A—C11A	121.7 (2)	C11B—C12B—C13B	121.7 (2)
C13A—C12A—H12A	119.2	C11B—C12B—H12B	119.1
C11A—C12A—H12A	119.2	C13B—C12B—H12B	119.1
C12A—C13A—C8A	118.18 (17)	C8B—C13B—C12B	117.96 (18)
C12A—C13A—C14A	117.78 (17)	C8B—C13B—C14B	123.96 (16)
C8A—C13A—C14A	123.85 (16)	C12B—C13B—C14B	117.86 (17)
O1A—C14A—C1A	106.40 (14)	O1B—C14B—C1B	106.49 (15)
O1A—C14A—C13A	109.58 (15)	O1B—C14B—C15B	108.00 (14)
C1A—C14A—C13A	105.90 (14)	C1B—C14B—C15B	110.53 (14)
O1A—C14A—C15A	108.06 (14)	O1B—C14B—C13B	109.25 (14)
C1A—C14A—C15A	110.73 (14)	C1B—C14B—C13B	105.96 (14)
C13A—C14A—C15A	115.78 (14)	C15B—C14B—C13B	116.21 (16)
C16A—C15A—C14A	116.41 (15)	C16B—C15B—C14B	116.08 (16)
C16A—C15A—H15A	108.2	C16B—C15B—H15C	108.3
C14A—C15A—H15A	108.2	C14B—C15B—H15C	108.3
C16A—C15A—H15B	108.2	C16B—C15B—H15D	108.3
C14A—C15A—H15B	108.2	C14B—C15B—H15D	108.3
H15A—C15A—H15B	107.3	H15C—C15B—H15D	107.4
C17A—C16A—C15A	115.75 (16)	C17B—C16B—C15B	116.37 (17)
C17A—C16A—H16A	108.3	C17B—C16B—H16C	108.2
C15A—C16A—H16A	108.3	C15B—C16B—H16C	108.2
C17A—C16A—H16B	108.3	C17B—C16B—H16D	108.2
C15A—C16A—H16B	108.3	C15B—C16B—H16D	108.2
H16A—C16A—H16B	107.4	H16C—C16B—H16D	107.3
N1A—C17A—C16A	113.87 (15)	N1B—C17B—C16B	114.20 (16)
N1A—C17A—H17A	108.8	N1B—C17B—H17C	108.7
C16A—C17A—H17A	108.8	C16B—C17B—H17C	108.7
N1A—C17A—H17B	108.8	N1B—C17B—H17D	108.7
C16A—C17A—H17B	108.8	C16B—C17B—H17D	108.7
H17A—C17A—H17B	107.7	H17C—C17B—H17D	107.6
N1A—C18A—H18A	109.5	N1B—C18B—H18D	109.5
N1A—C18A—H18B	109.5	N1B—C18B—H18E	109.5
H18A—C18A—H18B	109.5	H18D—C18B—H18E	109.5
N1A—C18A—H18C	109.5	N1B—C18B—H18F	109.5
H18A—C18A—H18C	109.5	H18D—C18B—H18F	109.5
H18B—C18A—H18C	109.5	H18E—C18B—H18F	109.5
N1A—C19A—H19A	109.5	N1B—C19B—H19D	109.5
N1A—C19A—H19B	109.5	N1B—C19B—H19E	109.5
H19A—C19A—H19B	109.5	H19D—C19B—H19E	109.5
N1A—C19A—H19C	109.5	N1B—C19B—H19F	109.5
H19A—C19A—H19C	109.5	H19D—C19B—H19F	109.5
H19B—C19A—H19C	109.5	H19E—C19B—H19F	109.5
			
C6A—C1A—C2A—C3A	1.2 (3)	C6B—C1B—C2B—C3B	−1.7 (3)
C14A—C1A—C2A—C3A	178.23 (17)	C14B—C1B—C2B—C3B	179.50 (17)
C1A—C2A—C3A—C4A	−1.2 (3)	C1B—C2B—C3B—C4B	0.8 (3)
C2A—C3A—C4A—C5A	−0.2 (3)	C2B—C3B—C4B—C5B	0.6 (3)
C3A—C4A—C5A—C6A	1.6 (3)	C3B—C4B—C5B—C6B	−1.0 (3)
C4A—C5A—C6A—C1A	−1.6 (3)	C4B—C5B—C6B—C1B	0.1 (3)
C4A—C5A—C6A—S1A	174.51 (16)	C4B—C5B—C6B—S1B	−176.25 (16)
C2A—C1A—C6A—C5A	0.2 (3)	C2B—C1B—C6B—C5B	1.2 (3)
C14A—C1A—C6A—C5A	−176.66 (16)	C14B—C1B—C6B—C5B	179.94 (17)
C2A—C1A—C6A—S1A	−175.12 (14)	C2B—C1B—C6B—S1B	176.89 (15)
C14A—C1A—C6A—S1A	8.0 (3)	C14B—C1B—C6B—S1B	−4.4 (3)
C7A—S1A—C6A—C5A	156.28 (14)	C7B—S1B—C6B—C5B	−153.88 (15)
C7A—S1A—C6A—C1A	−28.0 (2)	C7B—S1B—C6B—C1B	30.1 (2)
C6A—S1A—C7A—C8A	−29.24 (17)	C6B—S1B—C7B—C8B	22.7 (2)
S1A—C7A—C8A—C9A	−114.43 (17)	S1B—C7B—C8B—C13B	−63.9 (2)
S1A—C7A—C8A—C13A	67.3 (2)	S1B—C7B—C8B—C9B	118.40 (19)
C13A—C8A—C9A—C10A	1.4 (3)	C13B—C8B—C9B—C10B	−2.9 (3)
C7A—C8A—C9A—C10A	−176.99 (18)	C7B—C8B—C9B—C10B	174.94 (19)
C8A—C9A—C10A—C11A	−0.3 (3)	C8B—C9B—C10B—C11B	0.2 (3)
C9A—C10A—C11A—C12A	−0.6 (3)	C9B—C10B—C11B—C12B	2.2 (3)
C10A—C11A—C12A—C13A	0.3 (3)	C10B—C11B—C12B—C13B	−1.9 (3)
C11A—C12A—C13A—C8A	0.7 (3)	C9B—C8B—C13B—C12B	3.1 (3)
C11A—C12A—C13A—C14A	−174.38 (16)	C7B—C8B—C13B—C12B	−174.58 (17)
C9A—C8A—C13A—C12A	−1.6 (3)	C9B—C8B—C13B—C14B	−171.44 (17)
C7A—C8A—C13A—C12A	176.71 (17)	C7B—C8B—C13B—C14B	10.9 (3)
C9A—C8A—C13A—C14A	173.24 (17)	C11B—C12B—C13B—C8B	−0.7 (3)
C7A—C8A—C13A—C14A	−8.5 (3)	C11B—C12B—C13B—C14B	174.11 (17)
C2A—C1A—C14A—O1A	0.3 (2)	C2B—C1B—C14B—O1B	−1.8 (2)
C6A—C1A—C14A—O1A	177.21 (16)	C6B—C1B—C14B—O1B	179.50 (16)
C2A—C1A—C14A—C13A	−116.21 (17)	C2B—C1B—C14B—C15B	−118.81 (18)
C6A—C1A—C14A—C13A	60.7 (2)	C6B—C1B—C14B—C15B	62.4 (2)
C2A—C1A—C14A—C15A	117.54 (18)	C2B—C1B—C14B—C13B	114.50 (18)
C6A—C1A—C14A—C15A	−65.6 (2)	C6B—C1B—C14B—C13B	−64.2 (2)
C12A—C13A—C14A—O1A	−11.1 (2)	C8B—C13B—C14B—O1B	−176.78 (16)
C8A—C13A—C14A—O1A	174.06 (15)	C12B—C13B—C14B—O1B	8.7 (2)
C12A—C13A—C14A—C1A	103.27 (18)	C8B—C13B—C14B—C1B	68.8 (2)
C8A—C13A—C14A—C1A	−71.5 (2)	C12B—C13B—C14B—C1B	−105.66 (18)
C12A—C13A—C14A—C15A	−133.61 (17)	C8B—C13B—C14B—C15B	−54.3 (2)
C8A—C13A—C14A—C15A	51.6 (2)	C12B—C13B—C14B—C15B	131.16 (17)
O1A—C14A—C15A—C16A	−76.66 (19)	O1B—C14B—C15B—C16B	72.5 (2)
C1A—C14A—C15A—C16A	167.18 (15)	C1B—C14B—C15B—C16B	−171.34 (16)
C13A—C14A—C15A—C16A	46.6 (2)	C13B—C14B—C15B—C16B	−50.6 (2)
C14A—C15A—C16A—C17A	81.9 (2)	C14B—C15B—C16B—C17B	−84.3 (2)
C19A—N1A—C17A—C16A	161.24 (17)	C18B—N1B—C17B—C16B	73.7 (2)
C18A—N1A—C17A—C16A	−75.9 (2)	C19B—N1B—C17B—C16B	−163.21 (19)
C15A—C16A—C17A—N1A	−66.7 (2)	C15B—C16B—C17B—N1B	67.5 (2)

### Hydrogen-bond geometry (Å, °)

**Table:**

Cg1, Cg2, Cg7 and Cg8 are the centroids of the C1A—C6A, C8A—C13A, C1B–C6B and C8B—C13B rings, respectively.

**Table:**

*D*—H···*A*	*D*—H	H···*A*	*D*···*A*	*D*—H···*A*
O1A—H1A···N1A	0.84	1.86	2.693 (2)	170
O1B—H1B···N1B	0.84	1.84	2.679 (2)	174
C4A—H4AA···O1B	0.95	2.51	3.253 (2)	135
C3A—H3AA···Cg7^i^	0.95	2.74	3.526 (6)	140
C17A—H17A···Cg1^ii^	0.99	2.67	3.537 (7)	147
C17A—H17B···Cg2^ii^	0.99	2.75	3.720 (3)	167
C17B—H17C···Cg8^iii^	0.99	2.68	3.663 (6)	170
C17B—17D···Cg7^iii^	0.99	2.64	3.538 (1)	149

Symmetry codes: (i) −*x*+1, −*y*+1, −*z*+1; (ii) *x*+1, *y*, *z*; (iii) *x*−1, *y*, *z*.
